# Digital Health Competencies Among Health Care Professionals: Systematic Review

**DOI:** 10.2196/36414

**Published:** 2022-08-18

**Authors:** Jessica Longhini, Giacomo Rossettini, Alvisa Palese

**Affiliations:** 1 Department of Medical Sciences, University of Udine Udine Italy; 2 School of Physiotherapy, University of Verona Verona Italy

**Keywords:** eHealth literacy, eHealth competencies, digital health, competencies, eHealth, health literacy, digital technology, health care professionals, health care workers, review, systematic review

## Abstract

**Background:**

Digitalization is not fully implemented in clinical practice, and several factors have been identified as possible barriers, including the competencies of health care professionals. However, no summary of the available evidence has been provided to date to depict digital health competencies that have been investigated among health care professionals, the tools used in assessing such competencies, and the effective interventions to improve them.

**Objective:**

This review aims to summarize digital health competencies investigated to date and the tools used to assess them among health care professionals.

**Methods:**

A systematic review based on the PRISMA (Preferred Reporting Items for Systematic Reviews and Meta-Analyses) checklist was performed. The MEDLINE, Cumulative Index to Nursing and Allied Health Literature, PsycINFO, and Scopus databases were accessed up to September 4, 2021. Studies assessing digital health competencies with quantitative designs, targeting health care professionals, and written in English were included. The methodological quality of included studies was evaluated using the Joanna Briggs Institute tools.

**Results:**

A total of 26 studies, published from 1999 to 2021, met the inclusion criteria, and the majority were cross sectional in design, while only 2 were experimental study designs. Most studies were assessed with moderate to low methodological quality; 4 categories and 9 subcategories of investigated digital health competencies have been identified. The most investigated category was “Self-rated competencies,” followed by “Psychological and emotional aspects toward digital technologies,” “Use of digital technologies,” and “Knowledge about digital technologies.” In 35% (9/26) of the studies, a previously validated tool was used to measure the competencies assessed, while others developed ad hoc questionnaires.

**Conclusions:**

Mainly descriptive studies with issues regarding methodology quality have been produced to date investigating 4 main categories of digital health competencies mostly with nonvalidated tools. Competencies investigated might be considered while designing curricula for undergraduate, postgraduate, and continuing education processes, whereas the methodological lacks detected might be addressed with future research. There is a need to expand research on psychological and emotional elements and the ability to use digital technology to self-learn and teach others.

**Trial Registration:**

PROSPERO International Prospective Register of Systematic Reviews CRD42021282775; https://www.crd.york.ac.uk/prospero/display_record.php?RecordID=282775

## Introduction

### Background

Over the last few decades, the increasing technology development has led to a wide digitalization of several work processes in health care settings. The World Health Organization (WHO) has recently defined and categorized digital health interventions in the health care context as “a discrete function of the digital technology to achieve health care sector objectives” [1]. The framework developed by the WHO includes a wide range of digital tools and interventions, such as telemonitoring, the use of artificial intelligence, decision-making algorithms, and health data collection [1]. According to the evidence available, digitalization has improved the quality of care, affecting several outcomes at the system level (eg, safety in medication administration and length of in-hospital stay) and at the individual level (eg, increasing functional/cognitive abilities and patients’ satisfaction) [2].

Despite its potential effectiveness, digitalization is not fully implemented in clinical practice. Several factors have been identified as possible barriers, including the availability of technology, financial resources, and health care professionals’ skills in using digital technology [3]. To improve health care digitalization, health professionals have been recognized as a key factor in the digital transformation of the health care sector. Therefore, they should be equipped with digital health competencies, from basic (eg, computers, tablets) to more complex skills, such as teaching patients about the safe and appropriate use of digital data sources and technology [3].

### Digital Health Competencies

Different terms have been established to date by the literature to refer to digital health competencies. The most common term is eHealth literacy, which has been defined as the ability to use information retrieved from an electronic source to solve a health problem [4]. Conceptual frameworks describing the concept and components of eHealth literacy have been developed to date for citizens and patients [5]. For example, Norman and Skinner’s Lily framework [4] includes 6 literacy competencies, namely, health, traditional, information, scientific, computer, and media literacy. These competencies have been further expanded, with updated frameworks such as the “Patient Readiness to Engage in Health Internet Technology” (PRE-HIT) and the “eHealth Literacy Framework” (eHLF). These include different elements promoting or hindering eHealth literacy such as motivation, engagement, willingness, anxiety, expectations, and beliefs [6,7]. However, the concepts and components considered in these frameworks should be conceived differently when referring to health care professionals, given that they are expected to have the competencies required to solve patients’ problems rather than a personal health problem [8]. As a result of this gap, and in light of the required competencies to overcome barriers in health care digitalization processes [3], an emergent area of investigation has been set around the digital health competencies of health care professionals.

Different frameworks have been developed also in this context, mostly targeting a specific profession, mainly nurses, and using the methodology of expert consultation, surveys, and consensus (eg, the Delphi study) [5]. Among the most recent frameworks, the Health Information Technology Competencies (HITCOMP) [9] framework and the Technology Informatics Guiding Education Reform (TIGER) version 2.0 framework [10] have both identified 33 areas of competence articulated in domains. Specifically, the HITCOMP framework [9] has provided 5 domains, namely, (1) administration, (2) research/biomedicine, (3) direct patient care, (4) informatics, and (5) engineering/information systems/information and communications technology (ICT).

The TIGER framework has described relevant competencies [10] for those who provide direct patient care, including communication, documentation, quality and safety management, teaching, training/education, and ethics in health information technology [10].

In this context, a recent review dared to summarize the digital health competencies expected by health care professionals by synthetizing 30 available frameworks [5]. According to the findings, discrepancies and overlapping are still present across available frameworks regarding the different categorization of the competencies, the methods used to conceptualize such frameworks, and the competencies included [5]. These inconsistencies rely on the different health care professions targeted, including health professionals not involved in direct care, such as engineers [10]. Moreover, half of the 30 frameworks [5] emerged from gray literature and 30% were developed with the involvement of students, thus with different expected responsibilities and competencies [5].

Furthermore, the development of the digital health competencies according to the emergence of new technologies requires a continuous updating of both competencies to consider relevant and methods to assess appropriately these competencies [5]. However, to the best of our knowledge, no recent systematic reviews have been performed on digital health competencies among health care professionals. Providing a systematic summary of literature might inform policymakers, managers, and educators about how to appropriately measure the level of competencies in health care sector and how to develop adequate training programs to fill the gap in the digital health competencies. Moreover, a summary of the available evidence may inform researchers about the gaps in this field of investigation. Therefore, this systematic review aims to summarize which digital health competencies have been studied in literature and with what tools they have been measured to date among health care professionals.

## Methods

### Research Questions

Two main research questions have been addressed: (1) Which digital health competencies have been investigated to date among health care professionals? (2) How have these competencies been assessed?

### Study Design

We conducted a systematic review by adopting the PRISMA (Preferred Reporting Items for Systematic Reviews and Meta-Analyses; Multimedia Appendix 1) checklist [11] both in protocol development and in method and finding reporting. The protocol has been submitted for evaluation to the International Prospective Register of Systematic Reviews (PROSPERO; registration number CRD42021282775).

### Eligibility Criteria

Studies satisfying the following criteria were included: (1) assessing digital health competencies as an umbrella term (thus including terms related and similar to, eg, digital literacy [12], health informatics competencies [10], or eHealth competencies [13,14]); (2) targeting health care professionals; (3) adopting a quantitative design (eg, randomized control trial, quasi-experimental trial, longitudinal, cross-sectional studies); and (4) written in English. Therefore, qualitative studies, commentaries, editorials, letters, PhD dissertations, conference abstracts, and all studies that investigated technology accessibility were excluded.

### Data Searching

The search string was designed and developed with the support of an expert research librarian and then preliminarily piloted in a database to ensure its accuracy according to the review aims. The final string search included the following keywords: (1) “digital competencies” and “eHealth literacy” in their similar and affiliated terms (eg, digital Health Literacy,” “digital literac*,” “digital competenc*,” “digital skill*”; and (2) “health professionals” in its affiliated and similar term (“health care practice*,” “nurs*”) as fully reported in Multimedia Appendix 2. The search string was applied in the following databases: MEDLINE, Cumulative Index to Nursing and Allied Health Literature, PsycINFO, and Scopus up to September 4, 2021, with an English language restriction filter. In addition, the “TITLE-ABS-KEY” filter was adopted for the SCOPUS database to detect relevant studies. The reference lists of the included studies, the available trial registries, and the references of systematic reviews were screened by hand-searching to retrieve all relevant studies. Moreover, Mendeley Reference Manager was used to manage all references and delete duplicates.

### Study Selection

The title, the abstract, and the full-text screening of eligible studies were performed by 2 researchers (JL and GR) independently, and disagreements were resolved by a third researcher (AP). Interrater reliability was assessed using Cohen κ statistics, and it resulted in a value of 0.83 (95% CI 0.73-0.93), meaning an almost perfect level of agreement [15].

**Figure 1:**
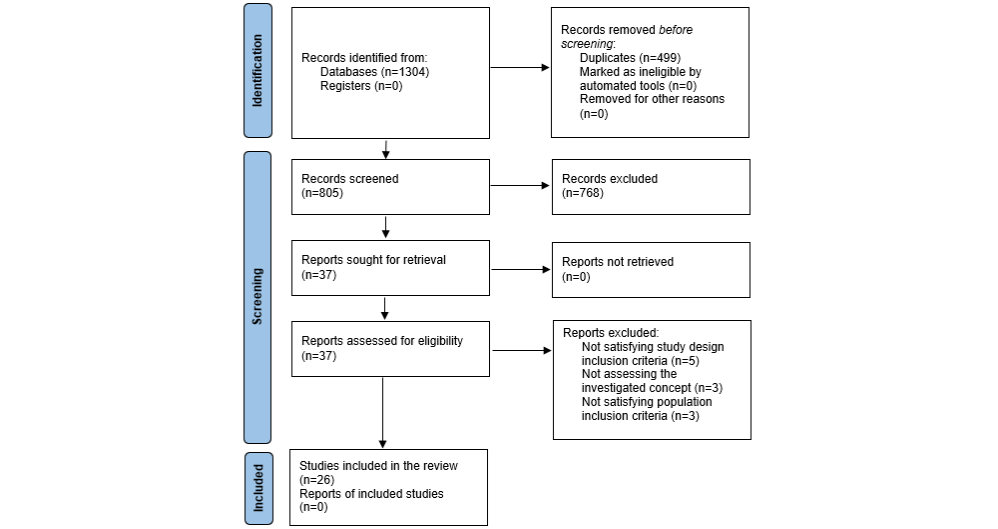
PRISMA (Preferred Reporting Items for Systematic Reviews and Meta-Analyses) flow diagram for new systematic reviews that included searches of databases and registers only (Page et al [11]).

The study selection process is summarized in Figure 1 according to the PRISMA flow diagram [11].

### Methodological Quality Assessment

Studies were assessed for their methodological quality by 2 researchers (JL and GR) independently, and a third researcher (AP) was consulted to resolve disagreements.

Joanna Briggs Institute tools for analytical cross-sectional [16], prevalence [17], and randomized control trial studies [18] were adopted according to the design used in the included studies. Specifically, regarding observational studies, we considered analytical cross-sectional studies when the statistical analysis was performed to identify associations between variables; otherwise they were considered prevalence studies [17].

For all quality assessment tools adopted, the scores applied were “Y” (yes) when the item was satisfied, “N” (no) when the item was not satisfied, and “U” (unclear) when the information contained in the study was not sufficient. Cut-off criteria were established through an agreement process among researchers based on previous evidence [19,20]. A moderate methodological quality level was identified when positive answers (= yes) were scored from 5 to 6 in analytical cross-sectional studies, from 6 to 7 in prevalence studies, and 10 or 11 in randomized control trials. Positive answers below and above these values were considered low and high methodological quality, respectively.

However, to comprehend all studies, their methodological quality was not considered an exclusion criterion.

### Data Extraction, Analysis, and Synthesis

The following data were extracted from each included study: author(s); year of publication; country; study design; population characteristics (eg, age, work profile) and number of participants; investigated digital health competencies; definition(s) provided of the assessed competencies (as reported in the “Study Background” or in the “Methods” sections); tools; and data collection methods used to assess the competencies investigated.

Two Excel grids were developed to extract data from the included studies according to the study aims. The grids were piloted among 3 studies to ensure their feasibility, and consistency was also assessed among the researchers (JL and GR) who performed the data extraction.

After having extracted the data, first, the study characteristics were summarized according to the study design (analytical cross-sectional, prevalence, and randomized control trial studies), reporting their main features and methodological quality. Second, following the aims of this systematic review, digital health competencies were summarized by extracting and analyzing items as open- or closed-ended questions included in the tools used to assess such competencies in each study, irrespective of their formats [21]. The items that emerged were grouped into categories and, when needed, into subcategories through a content analysis [22]. In the content analysis, the researchers adopted a systematic coding and categorizing approach to textual information extracted from the studies to merge patterns, and structure them into main categories and subcategories, by also reporting the frequency [23]. Two researchers (JL and GR) independently performed the entire process, and disagreements were resolved by a third researcher (AP). From the analysis of 362 extracted items used to assess digital health competencies in the included studies, 4 main categories emerged, namely, “Self-rated competencies,” “Psychological and emotional aspects toward the use of digital technologies,” “Use of digital technologies,” and “Knowledge about digital technologies.” Then, the number of items used across studies and the number of studies that assessed each specific category of competence were counted. Furthermore, tools used to assess the competencies in included studies were summarized into their main features.

## Results

### Main Characteristics of Studies Identified

A total of 1304 studies were identified from literature searches, of which 26 met the inclusion criteria (Figure 1). The majority were cross-sectional studies, of which 11 [24-34] were considered prevalence data studies, and 13 [35-47] as analytical cross-sectional studies (Table 1). Among the remaining ones, 2 were experimental studies [48,49]. The studies included were conducted over a wide range of years, from 1999 [25] to 2021 [27], and more than 65% (17/26) of them [26-28,32,33, 35,37,39,40,42,44] have been published in the last 5 years.

In total, 5 studies were conducted in the United States of America [25,30,38,40,41], while the others were performed in different European countries (eg, Germany [34,35] and Finland [25,26]), and in low-income countries (eg, Malawi [48] and Uganda [43]). In terms of the setting, 9 studies [32,33,36, 37,43-46,49] were conducted in hospitals. By contrast, the others were performed in mixed settings (eg, acute care [26], local health departments [25], and community [48]).

A total of 8 studies [26,32,36,38,41,42,47,49] involved nurses and 7 [25,27,28,33,39,44,45] covered health care professionals, while the others involved specific roles (eg, psychiatrists [40], pharmacists [31], maternal and child professionals [30]; Tables 1 and 2). The sample size was variable across the studies, ranging from 36 [30] to 5209 participants [39] with a variable age mostly comprised between 30 [46] and 50 years [27].

**Table 1:** Characteristics of included analytical cross-sectional and prevalence studies.

Study type and reference		Country	Study design	Setting(s)	Sample and profession; age	Competencies assessed	Definition provided of the competencies assessed	Tools/data collection method(s) and items
**Analytical cross-sectional studies**								
	Campbell and McDowell [38]	United States	Descriptive	Community hospital (100 beds)	112 registered nurses; 35 (31.2%) born in the 1960s	Self-perceived computer literacy	Computer literacy: “the skills necessary for accessing and using information, managing files, navigating an operating system, and using common applications, such as word processing” (source: “Background” section)	Gassert/McDowell Computer Literacy Survey (15 items)
	Do et al [39]	Vietnam	Cross sectional	12 hospitals and 3 health centers	5209 HCPs^a^, 905 (17.4%) aged between 41 and 60 years	eHealth literacy	N/A^b^	eHEALS^c^ questionnaire [15] (8 items to measure consumers’ combined knowledge, comfort, and perceived skills at finding, evaluating, and applying eHealth information to health problems)
	Duffy et al [40]	United States	Cross sectional	Mixed settings	152 psychiatrists; 67 (44 %) aged between 50 and 64 (mean 56.9) years	Comfort in using computers and other electronic devices for professional, personal, and clinical aims Computer use for specific clinical tasks	N/A	Web-and-paper-based survey (open- and closed-ended questions)
	Elhadi et al [37]	Libya	Cross sectional	Hospitals	673 specialists/senior physicians, physician trainees; 442 (65.7%) aged between 30 and 40 years	Using computer ability Awareness, knowledge, attitude, and computer skills about telemedicine	Awareness: N/A Knowledge: N/A Attitude: N/A Computer skills: level of “information technology and computer skills” (source: “Methods” section)	AKAS^d^ questionnaire [27] (Awareness, 12 items; Knowledge, 11 items; Attitude, 11 items; information technology/computer skills, 13 items)
	Gaumer et al [41]	United States	Cross sectional	Mixed settings	241 nurse practitioners; N/A	Use of information technology (general and for specific function) Benefits perceived from using technology (caregiving, time saving, patient safety) Self-perceptions about information technology competence	N/A	Questionnaire: Use of information technology (general: 1 item, specific functions: N/A) Perceived benefit, 3 items Self-perceptions about information technology competence, 1 item
	Gürdaş Topkaya and Kaya [36]	Turkey	Cross sectional	Hospitals	688 nurses; 293 (42.6%) aged between 20 and 29 years	Computer literacy and attitudes toward computers in health care	Computer literacy: “briefly defined as the ability to use a computer” as well as “the ability to control [a] computer in achieving certain goals,” “to use different computer applications,” “to comprehend [the] economic, psychological and social effects of computer[s] on [the] individual and society,” and “to use [a] computer [for] access to information, [for] communication and [in the] problem solution process” (source: “Background” section)	Multicomponent Assessment of Computer Literacy, 24 items Pre-test for Attitudes Towards Computers in Healthcare Assessment Scale version 2, 40 items
	Hennemann et al [35]	Germany	Cross sectional	Rehabilitation facilities	149 participants (nurses, psychologists, physical therapists, physicians, patient administration, social workers, art/body/occupational therapists, nutritionists, medical technical assistants); mean 44.35 (SD 11.27) years	Acceptance of eHealth intervention and of online aftercare Information technology literacy eHealth literacy Performance expectancy Effort expectancy Internet anxiety Knowledge of eHealth interventions	Acceptance (operationalized according to the UTAUT^e^) “the intention to use eHealth interventions for patients’ health promotion in work context, and adoption of online aftercare” eHealth literacy: the ability to find, evaluate, and utilize internet-based health information to health problems” (source for both: “Methods” section) N/A for others	Self-administered web-based questionnaire (acceptance, 4 items; information technology literacy, 1 item; performance expectancy, 2 items; effort expectancy, 2 items; internet anxiety, 2 items; knowledge of eHealth interventions, 2 items) eHEALS questionnaire [15] (8 items)
	Kritsotakis et al [42]	Greece	Cross sectional	Secondary and primary general-care hospitals	200 nurses and nursing assistants; 70 (35%) aged 45-54 years	eHealth literacy	“The ability to find and assess health-related information online at the individual level” (source: “Methods” section)	eHEALS questionnaire [15] (8 items)
	Olok et al [43]	Uganda	Cross sectional	Hospitals	68 doctors; 33 (48.5%) aged 31-40 years	Attitudes toward eHealth Level of ICT^f^ use and skills	N/A	Questionnaire: internal consistence evaluated (level of ICT use and skills on the same 18 items—list of facilities and tools; attitudes, 25 items divided into relative advantages, compatibility, complexity, trialability—not considered, observability)
	Shiferaw and Mehari [44]	Ethiopia	Cross sectional	Hospital	287 HCPs; mean 30.09 (SD 5.025) years	Internet use (types and frequency) eHealth literacy	Internet use: “Health professionals’ practice of using the Internet for browsing health-related information to make sound decisions” eHealth literacy: “participants’ ability to locate and use credible information from the Internet” (source: “Methods” section)	Internet use, 15 items eHEALS questionnaire [15] (8 items)
	Tesfa et al [45]	Ethiopia	Cross sectional	Teaching hospitals	383 HCPs (nurses, doctors, midwives, pharmacists, laboratory technicians); mean 28.3 (SD 3.37) years	Electronic health information resource utilization (information searching, technical skills) and purpose of use Computer literacy eHealth literacy Awareness Attitude Motivational factors (perceived usefulness and use)	N/A	Questionnaire (purpose of use, 5 items; N/A for others) eHEALS questionnaire [15] (8 items)
	Thapa et al [46]	Saudi Arabia	Cross sectional	Hospitals	218 physicians and nurses; 61 (28%) aged between 31 and 35 years	Willingness to use digital health tools in patient care Attitudes and self-efficacy toward using digital health tools Digital health tools use perceived benefits and costs	Willingness: N/A Self-efficacy: “The belief in one’s own ability to successfully perform various specific actions related to the use of digital tools in patient care” Attitude: “The perceived relevance/value of different functions of digital tools for active engagement of patients in their own treatment/care” Perceived benefits: “Positive consequences of using digital tools” Perceived costs: “Potential psychological, financial, technological and administrative burden” (source: “Methods” section)	Questionnaire (willingness, 1 item; self-efficacy, 12 items; attitude, 10 items; perceived benefit and costs, 20 items)
	Vehko et al [47]	Finland	Cross sectional	Hospitals, primary care, private practice, social care, and others	3407 registered nurses; mean age 46.2 (SD 10.99) years	Nurses’ informatics competence: classification competence; e-care competence; e-documentation competence; ethics competence	Classification competence: “Planning, implementation and evaluation of care needs, and the use of the care process according to Finnish Care Classification” E-care competence: “Use of eHealth tools in tailoring patient care” E-documentation competence: “Electronic recording of patient data” E-ethics competence: “Competence in the ethical and safe way to use patient information systems” (source: “Methods” section)	Questionnaire (16 items)
**Prevalence studies**								
	Brady and Knox [24]	Northern Ireland	Cross sectional	N/A	98 psychiatric trainees/consultants (specialist registered, senior house officers, staff grades, consultants); age N/A	Self-rated computing skill levels	N/A	Questionnaire (6 items)
	Hollander and Martin [25]	United States	Cross sectional	344 local health departments	Some of or all public health professional staff working in the local health departments; age N/A	Staff internet use and resources used	N/A	Questionnaire (N/A)
	Kirchberg et al [34]	Germany	Cross sectional	N/A	93 physicians; 37 (40%) aged between 30 and 45 years	Level of knowledge of eHealth apps and data safety; mobile phone use; attitude toward (evaluation) medical apps for physician and patient use; evaluation of importance of medical app characteristics	N/A	Questionnaire (mobile phone use, 4 items; purpose of mobile phone use, 9 items, level of knowledge of eHealth apps and data safety, 9 items; evaluation of medical apps for physician use, list of 6 apps for patients and 5 apps for physicians; evaluation of importance of medical app characteristics, 7 items; evaluation of importance of privately used app characteristics, 7 items)
	Kleib and Nagle [26]	Canada	Cross sectional	Acute care, community, other settings	2844 nurses (generalist registered nurses and registered psychiatric nurses); 1257 (44%) aged ≥50 years	Use of patient care technology; self-perceived informatics competencies (foundational ICT skills, information and knowledge management, professional and regulatory accountability, and use of ICT in delivery of patient care)	Developed framework	Use of patient care technology (1 item) Self-perceived informatics competencies: Canadian Nurse Informatics Competence Assessment Scale (21 items)
	Kocher et al [27]	Switzerland	Explanatory sequential mixed method	University hospital, regional hospital, rheumatology outpatient clinics	47 professionals (registered nurses, physiotherapists, rheumatologists, occupational therapists, advanced practice nurses, general practitioners, psychologists, social workers, health policy); median age 41 (IQR 31-51) years	eHealth literacy (access, understand, appraise, apply)	eHealth literacy: “people’s knowledge, motivation and competence to ‘access’, ‘understand’, ‘appraise’ and ‘apply’ health information from electronic sources to address or solve a health problem” Access: “the ability to seek, find and obtain health information” Understand: “the ability to comprehend information” Appraise: “interpret and evaluate information” Apply: “the ability to use health information to make informed decisions” (source: “Background” section)	Questionnaire based on previously validated instruments (access, 6 items; understand and appraise, 5 items; apply, 9 items)
	Kujala et al [28]	Finland	Cross sectional	Public health organization	701 HCPs (nurses, social workers, physicians, dentists, ward secretaries, physiotherapists and other therapists, instrument or facility care personnel, health administration workers, psychologists); mean 44.1 (SD 11.9) years	Self-perceived eHealth competencies; actual patient guidance behaviors	eHealth competence: “A broad set of skills employing ICT and eHealth services, information management, multi-channel health coaching, patient communication, development and implementation” (source: “Background” section)	Questionnaire (self-perceived eHealth competencies, 9 items; actual patient guidance behaviors, 4 items)
	MacLure and Stewart [29]	Scotland	Cross sectional	Community and hospital pharmacies	94 participants (pharmacists, reregistration pharmacy graduates, pharmacy technicians, dispensing assistants, medicine counter assistants); 34 (36.2%) aged ≤29 years	Self-reported digital literacy	The British Computer Society defines digital literacy as “Being able to make use of technologies to participate in and contribute to modern social, cultural, political and economic life”. A similar definition of digital literacy is adopted in the United States: “the ability to use information and communication technologies to find, evaluate, create, and communicate information; it requires both technical and cognitive skills” (source: “Background” section)	Self-reported digital literacy (1 item)
	Polhamus et al [30]	United States	Cross sectional	N/A	36 maternal and child health professionals; 82% aged ≥40 years	Beliefs in the value of and confidence in using technology	Beliefs in the value of technology: “the extent to which they agreed with a set of questions about the value of a specific technology skill” (source: “Methods” section)	Questionnaire (beliefs, 3 items; confidence, 3 items)
	Thomas and Rutter [31]	England	Cross sectional	Mixed settings	386 pharmacists, 83 (21.5%) aged between 50 and 59 years	Confidence in basic computer skills and use of key software applications	N/A	Questionnaire (16 items)
	van Houwelingen et al [32]	The Netherlands	Cross sectional	Hospitals	1017 registered nurses; median age 41 (IQR 30-53) years	Confidence in their telehealth knowledge, skills, and attitudes	N/A	Questionnaire (31 items)
	Zayapragassarazan and Kumar [33]	India	Cross sectional	Teaching hospitals	120 Health professional faculty working; 57 (40%) aged between 30 and 40 years	Using computer ability; awareness, knowledge, attitude, and computer skills about telemedicine	N/A	AKAS questionnaire [27] (awareness, 12 items; knowledge, 11 items; attitude, 11 items; information technology and computer skills, 13 items)

^a^HCP: health care professional.

^b^N/A: not available.

^c^eHEALS: eHealth Literacy Scale.

^d^AKAS: Awareness, Knowledge, Attitude, Skills.

^e^UTAUT: Unified Theory of Acceptance and Use of Technology.

^f^ICT: information and communications technology.

**Table 2:** Characteristics of included randomized controlled trial studies.

Reference	Country	Study design	Setting	Sample and profession; age	Competence(s) assessed and definitions	Intervention	Tools/data collection method(s)
Jouparinejad et al [49]	Iran	Interventional study	Hospitals	60 nurses; 26 (43.3%) aged between 30 and 40 years	Nursing informatics competencies: Computer literacy: “The psychomotor skills to use computer tools, and knowledge of basic hardware and software functionality” Informatics literacy: “Nurses’ abilities to recognize, retrieve, evaluate and use information for patient care appropriately” Information management skills: “apply the data to support clinical decisions, documentation, data integrity, confidentiality and security” (source: “Methods” section)	Three-day workshop with theory and practice to develop nursing informatics competencies	Questionnaires: Adapted Nursing Informatics Competence Assessment Tool (30 items): computer literacy (10 items), informatics literacy (13 items), information management skills (7 items)
Mastellos et al [48]	Malawi	Randomized controlled trial	Community	40 community health professionals; 23/39 (49%) aged ≥40 years	Self-rated ICT^a^ knowledge; attitudes toward using computers, tablets, and smartphones	3-week blended learning “Introduction to ICT and eHealth” course (intervention) versus traditional course (control) on same contents	Questionnaire including 10 items to assess self-rated ICT knowledge, and 10 items to assess attitudes

^a^ICT: information and communications technology.

### Quality Assessment

The methodological quality was high in 5 analytical cross-sectional studies [35,39,42,46,47] (out of 12; Multimedia Appendix 3), in 1 prevalence study [26] (out of 11; Multimedia Appendix 4), and in 1 randomized control trial study [48] (out of 2; Multimedia Appendix 5). A total of 3 cross-sectional [38,41,43] and 7 prevalence studies [24,25,28-30,33,34] reported a low methodological quality. Among the former, no confounding factors were identified. By contrast, for all studies, the “Not applicable” option was assigned to the item regarding the use of “objective, standard criteria used for measurement of the condition.” Among the prevalence data studies, the most unclear item (10/11 studies) was regarding the adequacy of the sample size. By contrast, the item most often scored as “No” (5/11 studies) was the sample description.

### Digital Health Competencies Investigated

As many as 13/26 studies [27-30,35-38,42,44,46,47,49] reported the definitions of the concept assessed, which were retrieved from the “Methods” section in 8 studies [30,35,37,42,44, 46,47,49].

As summarized in Table 3, “Self-rated competencies” were assessed with 140 items grouped into 4 subcategories. “Digital literacy” emerged as the first subcategory in terms of frequency (59 items, 14 studies) and included items used to assess the self-perceived level of competence in using technology without a specific health goal (eg, in using tablets and mobile phones [43], apps [24], the internet [48], digital cameras [43], and computer literacy [45]). The second was the “eHealth literacy” subcategory, which included the 40 items provided by the 8-item eHealth Literacy Scale (eHEALS) [15] adopted by 5 studies [35,39,42,44,45]. Then, “Patient-oriented competencies” (21 items, 4 studies) included items aimed at assessing the ability, for example, to train and advise patients about technology [32], suitable websites [44], and apps [27], to create confidentiality, to maintain an ethical attitude and convey empathy through videoconferencing [28,32], and to assess the needs of patients regarding telehealth [32]. Lastly, the “Process of care-oriented competencies” subcategory (20 items, 11 studies) included those items assessing the level of competence in retrieving, evaluating, and applying online information, as well as in using eHealth tools to inform the decision-making process in patient care [26,47,49].

The second category, “Psychological and emotional aspects toward digital technologies,” was assessed with 110 items by 18 studies. The first subcategory, “Attitudes and beliefs” (82 items, 14 studies), included items assessing attitudes regarding the perceived benefits of the care delivered to and for patients (eg, quality of care and opportunity for self-care [46]); the work benefits perceived (eg, saving [41] and easy access to data [28]); the complexity [43]; the importance, value [28,30], and the feasibility in work [43] of using digital technologies and telemedicine [33,37]. Then, in the second subcategory, “Confidence” (21 items, 6 studies), most items were aimed at assessing the confidence in performing specific activities such as “searching the internet” [31] or “monitoring the patients’ health data using mobile apps” [46]. Finally, in the “Awareness” subcategory (7 studies, 4 items), items assessing the general level of awareness of telemedicine or health information resources and awareness meant as observability were included (eg, to observe the high use of information and communication technology in the workplace [43]).

The third category, “Use of digital technologies” (98 items, 13 studies), included the subcategory “General use of digital technologies” (51 items, 9 studies), which was adopted to investigate the extent to which health care professionals applied the digital technologies in general, for example, the use of computers, printers, the internet, email, and the “Use of digital technologies for specific functions” (47 items, 7 studies) for investigating specific functions as, for example, in documenting care [41], communicating with patients [40], or for research purposes [45].

Lastly, the fourth category, “Knowledge about digital technologies” (14 items, 5 studies), included items aimed at assessing knowledge regarding, for example, telemedicine [37], technical aspects [34], data protection and privacy requirements [32], security, and appropriateness of communication application (eg, WhatsApp, medCrowd) [34].

**Table 3:** Investigated areas of digital health competencies.

Category and subcategories		Item examples and references	Items, n (n=362)	Studies, n
**Self-rated competencies**			140	19
	Digital literacy	Self-rated level of computer skill on the application PowerPoint [43] Level of skills in using body scanner [43]	59	14
	eHealth literacy	8-item eHEALS^a^ tool [15]	40	5
	Patient-oriented competencies	“Can put patients at ease when they feel insecure about using technology?” [32] “Do you recommend apps to your patients that support them in a healthy lifestyle?” [28]	21	4
	Process of care-oriented competencies	“Can combine my nursing knowledge and experience effectively when using telehealth technology and decision-making” [32] “I am able to recognize (at a distance) the needs of the patient and determine the care situation” [32]	20	11
**Psychological and emotional aspects toward the use of digital technologies**			110	18
	Attitudes and Beliefs	“I believe that using ICT^b^ is cumbersome” [43] “Using ICT is compatible with all aspects of my work” [43] “Be a better caregiver by using information technology” [41]	82	14
	Confidence	“I believe I would be able to use a computer or mobile app to provide patient care” [48] “Confidence using the Internet logging on” [31]	21	6
	Awareness	“Awareness of telemedicine” [37] “ICT is very visible in the hospital where I work” [43]	7	4
**Use of digital technologies**			98	13
	General use of digital technologies	“Do you use and own a mobile phone?” [34] “If you use the internet, how frequently do you use it?” [44]	51	9
	Use of digital technologies for specific functions	“Do you use the Internet regularly for medical/professional updates?” [44] Using a computer for a specific clinical task: “Access online patient educational materials” [40]	47	7
**Knowledge about digital technologies**		“Is it appropriate to use common email for professional communication in health systems?” [34] “Do you think a legal obligation for external certification of medical apps is required?” [34]	14	5

^a^eHEALS: eHealth Literacy Scale.

^b^ICT: information and communications technology.

### Tools Used to Assess Digital Health Competencies

In 9/26 studies [26,35-39,42,44,45], previously developed and validated tools were adopted to self-assess the competencies, with 5 studies [35,39,42,44,45] reporting the use of the e-HEALS tool [15], while the remaining used the Gassert/McDowell Computer Literacy Survey [38], the Canadian Nurse Informatics Competency Assessment Scale [26], the Awareness, Knowledge, Attitude, Skills tool [37], the Multicomponent Assessment of Computer Literacy, and the Pre-test for Attitudes Towards Computers in Healthcare Assessment tools [36]. The authors of the other studies developed ad hoc questionnaires, using 1 (eg, [32]) or multiple (eg, [49]) questionnaires with the number of items ranging from 1 [29] to 47 [33], mainly including several general dimensions (eg, Awareness, Self-efficacy, Attitudes) [32,33]. In most studies, tools were described in detail by reporting the dimensions of competencies under evaluation and the number of items; only in a few studies was the description poor (eg, [25]; Tables 1 and 2).

## Discussion

The discussion has been developed under 2 main lines: around the principal findings emerged and the comparison of evidence emerged with available studies, by including in each the future directions recommended for both practice and research in this field.

### Principal Findings

Despite the increased relevance of digital health competencies among health care professionals [50], in the last 20 years, only a few studies have been published, slightly more than 1 per year, with an increase in the last 5 years. Moreover, although there is an urgent need to equip health care professionals with appropriate competencies given the progressive digitalization [1], most studies available to date are cross sectional or prevalence in design and only 2 are experimental studies. In addition, a few studies have been conducted with high methodological quality, suggesting improvements in this research field.

Studies available have been conducted in developed (eg, United States, Europe) and developing (eg, Uganda) countries where different health digital transformations are in place. Therefore, our findings may help policymakers and educators to set competencies according to the stage of digitalization experienced regarding the infrastructures available. However, roughly half of the studies have been focused on hospitals, whereas the community settings and districts have been involved to a lesser extent despite their increased need to implement digitalization with competent health care professionals to address emerging inequalities and issues in terms of health care accessibility [51]. Moreover, studies have more often involved nurses, doctors, or mixed samples of health care professionals, suggesting that all health care profiles have been involved to date, albeit to a limited extend for some (eg, physiotherapists [8]). Given the progressive and expansive permeation of digitalization in the health care sector, all health care professionals should be involved in the assessment of digital health competencies aimed at tailoring educational strategies. Meriting attention is the variable age of participants involved in the studies, from new graduates to mature health care professionals close to retirement. The new generations, also called the “digital native generation” [52], have more attitudes toward digitalization [53], and this suggests the need to deepen this area of study by investigating in future studies specific digital health competencies, despite including other elements such as attitudes (eg, using a computer) that might be relevant only among mature health care professionals.

At the overall level, only half of the studies [27-30,35-38,42,44,46,47,49] reported the definitions of the competencies assessed, and these have been reported mainly in the “Methods” and “Background” sections.

This finding suggests that future studies should be strengthened in their conceptualization and grounded in their development on clear conceptual frameworks and definitions.

Four main categories of investigated areas regarding digital health competencies have emerged, along with 9 subcategories. The area most investigated to date is self-rated competencies, in line with available literature [4,9,10]. In particular, this area includes, among the others, competencies aimed at solving patients’ health or care plan issues. This point suggests an interest among the scientific community in investigating these competencies from innovative perspectives. Training, advising, and supporting patients in the appropriate and confident use of technologies and information retrieved from different ICTs, social media, and internet sources are crucial [54], as also underlined by the framework recently developed by a consortium of multiple European countries [14]. The interest in investigating psychological and emotional aspects of the use of digital technologies has grown increasingly over the years, being assessed in 18 studies. The perceived usefulness for smoothing the care processes, improving its quality and patient satisfaction, and understanding health conditions and the adherence to treatments are crucial elements. Attitudes, acceptance, and confidence [8,12] in using digital technologies, such as electronic prescriptions, remote monitoring, and electronic databases, have demonstrated a positive effect on care processes and patients’ outcomes [2].

A limited number of studies have investigated the use of and the knowledge regarding digital technologies. However, a review of frameworks on digital health competence identified these topics in almost 60% of them [5], suggesting an evident need to promote the awareness of these issues in future research, given the increasing threats to data safety from illegal hacking [55].

A lack of validated tools to measure digital health competencies has emerged. One-third of studies have used a validated tool, the eHEALS of Norman and Skinner [15], although it was developed for patients, thus requiring a specific validation process and adaptation in the field of health care professionals. Moreover, a propensity to develop ad hoc instruments rather than using those already validated has emerged. The reasons for this may rely on the limitations perceived by those available, as well as the rapid evolution of digital technologies and instruments that may require a continuous updating of the competencies to assess. Moreover, in all studies, the tools were intended to assess the perceptions of health care professions rather than measuring their digital health competencies objectively. Self-rated competencies might be useful while educational needs are investigated; however, the actual performance requires objective measurement systems that should be developed in this field.

### Comparison With Prior Work

Comparing the categories of competencies emerged in available studies with frameworks established in this field might inform the future directions in both educational practice and research. At the overall level, similarities and divergences emerged. The most common competence between previous frameworks [5,7] and that emerged in our study included the technical skills and the ability to manage and understand information retrieved from technology, including the internet. Psychological and emotional aspects were also highly investigated [4,10,12,14] among the studies included in this review in line with Norgaard and colleagues’ [7] eHLF for eHealth users. The engagement, the ability to take responsibility, the perception of feeling safe, and motivation were part of the framework as elements expressing the interaction between the person and the system [7]. Therefore, a debate on how these aspects may influence the digital health competence among health care professionals as well as how to transform them into professional competencies to evaluate merits further consideration.

A recent review indicated that most interventions that aimed to improve the digital health competencies of health care professionals focused on the capability rather than motivation in using eHealth [56]. Interventions promoting digital health competencies should also consider social and environmental factors, foreseeing participatory approaches, to bolster also the emotional and psychological factors toward the use of technology [56]. On the other side, discrepancies emerged regarding teaching, self-development, and learning abilities [12]. The National Health Service (NHS) framework on digital capability [12] embeds domains regarding the abilities, for example, to use digital technologies for personal learning and teaching others [12]. No similar elements emerged in our review. Therefore, future research should focus on the measurement of competencies regarding those aspects, while also considering increased use of blended learning and massive online open courses in continuing education [57].

As highlighted by a previous review [56], we also found that the competencies investigated are still mainly focused on health care professionals’ perspectives. However, increased attention is required when considering the competencies to assess patients’ needs, attitudes, barriers, facilitators, and potential benefits of being trained by health care professionals in the safe and appropriate use of technology and electronic information for health issues [47]. Therefore, from a self-perceived competence assessment mainly concerning general issues, efforts should now be addressed at developing patient-centered digital health care assessment tools capable of detecting all specific competencies involved in the entire process.

### Strengths and Limitations

This systematic review has several limitations. First, despite the accuracy of the process preventing the risk of publication bias by screening 4 databases and the reference lists of the included studies, as well as the trial registries [58], some studies may have been missed given that we adopted the English language filter and gray literature has not been searched.

Second, we adopted “digital health competencies” as an umbrella term to refer to all concepts that emerged from the literature. Although the use of all possible terms (eg, “digital health literacy”) in the search string and the inclusion process might have ensured inclusiveness, the summary provided under the same umbrella term might have introduced some limitations. Different aspects of digital health competencies, such as confidence, self-efficacy, attitude, and beliefs regarding digital technologies, have been considered relevant as affecting their use and appropriate adoption in the health care sector. Therefore, we included these elements as part of the umbrella term “digital health competencies,” relying on the previous frameworks including them [6,7]. This process has been considered a strength of this review because of the consideration of the full range of competencies as assessed in available studies. Third, previous frameworks [5] mainly focused on the categorization according to technical skills or functions (eg, safety management or care coordination); the content analysis [22,23] performed allowed to include all competencies as documented in retrieved studies, not limiting them to just skills and behaviors. Therefore, we valued also self-concepts, values, personal traits, and motivation (eg, [43]) to map all factors involved. However, the content analysis conducted to categorize the competencies that emerged from included studies was performed by researchers with different backgrounds (eg, nursing, physiotherapy). Although carefully conducted and its reliability assessed with the interreliability rate, their interpretations might have influenced the final categorizations. Lastly, we have synthesized studies originating from different countries, thus differences in health care digitalization might affect the generalizability of the conclusion drawn on future directions for research and training of health care professionals. These should be targeted and adapted according to the characteristic of the countries by training health care professionals based on the technologies available at a local level.

### Conclusion

Digital health competence among health care professionals is a new field of research that exploded in the last 5 years. However, studies conducted to date are mainly descriptive and have some methodological quality issues, suggesting lines of improvement. Moreover, with the increased decentralization of the health care sector, more studies are required in community settings, involving a wide range of health care professionals to assess the differences and commonalities in the competencies possessed and tailor specific educational strategies. Furthermore, with the increased size of the digital native generation among health care workers, specific digital health competencies instead of general ones should be investigated.

The different areas of competencies investigated to date might be considered while designing curricula for undergraduate, postgraduate, and continuing education processes. From the perspective of researchers, these competencies may drive the development of competence assessment tools, given the lack of validated instruments in this field, identifying more objective measures in addition to those based on self-perception. Furthermore, researchers should consider moving attention from the self-rated technical competencies to those embodying a patient-centered digital health care approach and related aspects that might affect the use of digital technologies.

In future frameworks and measurement tools, digital health competencies should be considered as a multicomponent competence, not limited to the technical skill, but rather expanded toward elements that might affect them. As our review showed, confidence, attitudes, beliefs, and awareness have been studied with increasing interest, suggesting the need to explore the relationships between different elements and understand how to train health care professionals properly. Curricula embedding the development of technical skills, knowledge, and psychological and emotional aspects of digital technology are recommended.

## Appendix

Multimedia Appendix 1 The Preferred Reporting Items for Systematic Reviews and Meta-Analyses checklist [11].

Multimedia Appendix 2 Search strings according to searched databases.

Multimedia Appendix 3 Quality for analytical cross-sectional studies [16].

Multimedia Appendix 4 Quality assessment for prevalence data studies [17].

Multimedia Appendix 5 Quality assessment for randomized control trial [18].

## Abbreviations

AKAS: Awareness, Knowledge, Attitude, Skills
eHEALS: eHealth Literacy Scale
eHLF: eHealth Literacy Framework
HCP: health care professional
HITCOMP: Health Information Technology Competencies
ICT: information and communications technology
N/A: not available
NHS: National Health Service
PRE-HIT: Patient Readiness to Engage in Health Internet Technology
PRISMA: Preferred Reporting Items for Systematic Reviews and Meta-Analyses
PROSPERO: International Prospective Register of Systematic Reviews
TIGER: Technology Informatics Guiding Education Reform
UTAUT: Unified Theory of Acceptance and Use of Technology
WHO: World Health Organization
