# Correction: Digital Health Competencies Among Health Care Professionals: Systematic Review

**DOI:** 10.2196/43721

**Published:** 2022-11-29

**Authors:** Jessica Longhini, Giacomo Rossettini, Alvisa Palese

**Affiliations:** 1 Department of Medical Sciences University of Udine Udine Italy; 2 School of Physiotherapy University of Verona Verona Italy

In “Digital Health Competencies Among Health Care Professionals: Systematic Review” (J Med Internet Res 2022;24(8):e36414) the author of a study included in our review, namely “Kocher, A, et al (2021). Patient and healthcare professional eHealth literacy and needs for systemic sclerosis support: a mixed methods study. RMD open, 7(3), e001783” noted two errors:

1. In the “Main Characteristics of Studies Identified” sub-section of “Results”, the maximum age of participants of studies included was incorrectly reported:

The sample size was variable across the studies, ranging from 36 [30] to 5209 participants [39] with a variable age range from 20 [36] to 68 years [27].

Thus, the sentence has been updated as follows:

The sample size was variable across the studies, ranging from 36 [30] to 5209 participants [39] with a variable age mostly comprised between 30 [46] and 50 years [27].

2. In Table 1, under the column “Sample and profession; age”, referring to the study of Kocher et al, 2021 [27], the age of professionals was incorrectly reported:

47 professionals (registered nurses, physiotherapists, rheumatologists, occupational therapists, advanced practice nurses, general practitioners, psychologists, social workers, health policy); median age 60 (IQR 50-68) years

Thus, the sentence has been updated as follows:

47 professionals (registered nurses, physiotherapists, rheumatologists, occupational therapists, advanced practice nurses, general practitioners, psychologists, social workers, health policy); median age 41 (IQR 31-51) years

The correction will appear in the online version of the paper on the JMIR Publications website on November 29, 2022, together with the publication of this correction notice. Because this was made after submission to PubMed, PubMed Central, and other full-text repositories, the corrected article has also been resubmitted to those repositories.

