# Supplementary material for: Digital Health Competencies Among Health Care Professionals: Systematic Review
Source: J Med Internet Res. 2022 Aug 18;24(8):e36414. doi: 10.2196/36414 (PMC9437781; doi:10.2196/36414)
Supplement: Multimedia Appendix 2 [file jmir_v24i8e36414_app2.docx]

**Multimedia Appendix 2. Search strings according to searched databases.**

| **Pubmed**  (“Digital Health Literacy” OR “eHealth literacy” OR e-literacy OR “digital literac*” OR “digital competenc*” OR “digital skill*” OR “technological skill*” OR “Technology competenc*” OR “media competenc*” OR “media skill*” OR “media literacy*” OR “technology skill*” OR “telehealth competenc*” OR “telehealth skill*” OR “telemedicine skill*” OR “telemedicine competenc*”) AND (“health professional*” or “healthcare practice*” OR nurs* OR physician* OR doctor* OR physioterap* OR “health work*”) |
| --- |
| **Total references retrieved 458** |
| **Cumulative Index to Nursing and Allied Health Literature**  (“Digital Health Literacy” OR “eHealth literacy” OR e-literacy OR “digital literac*” OR “digital competenc*” OR “digital skill*” OR “technological skill*” OR “Technology competenc*” OR “media competenc*” OR “media skill*” OR “media literacy*” OR “technology skill*” OR “telehealth competenc*” OR “telehealth skill*” OR “telemedicine skill*” OR “telemedicine competenc*”) AND (“health professional*” or “healthcare practice*” OR nurs* OR physician* OR doctor* OR physioterap* OR “health worker*”) |
| **Total references retrieved 298** |
| **SCOPUS**  TITLE-ABS-KEY ( “Digital Health Literacy” OR “eHealth literacy” OR e-literacy OR “digital literac*” OR “digital competenc*” OR “digital skill*” OR “technological skill*” OR “Technology competenc*” OR “media competenc*” OR “media skill*” OR “media literacy*” OR “technology skill*” OR “telehealth competenc*” OR “telehealth skill*” OR “telemedicine skill*” OR “telemedicine competenc*” ) AND TITLE-ABS-KEY ( “health profession*” OR “healthcare practice*” OR nurs* OR physician* OR doctor* OR physioterap* OR “health work*” ) AND ( LIMIT-TO ( DOCTYPE , “ar” ) ) AND ( LIMIT-TO ( LANGUAGE , “English” ) ) |
| **Total references retrieved 368** |
| **PsycINFO**  (“Digital Health Literacy” OR “eHealth literacy” OR e-literacy OR “digital literac*” OR “digital competenc*” OR “digital skill*” OR “technological skill*” OR “Technology competenc*” OR “media competenc*” OR “media skill*” OR “media literacy*” OR “technology skill*” OR “telehealth competenc*” OR “telehealth skill*” OR “telemedicine skill*” OR “telemedicine competenc*”) AND (“health professional*” or “healthcare practice*” OR nurs* OR physician* OR doctor* OR physioterap* OR “health worker*”) |
| **Total references retrieved 180** |
| **Total references retrieved 1304** |
