# Supplementary material for: Digital Health Competencies Among Health Care Professionals: Systematic Review
Source: J Med Internet Res. 2022 Aug 18;24(8):e36414. doi: 10.2196/36414 (PMC9437781; doi:10.2196/36414)
Supplement: Multimedia Appendix 3 [file jmir_v24i8e36414_app3.docx]

**Multimedia Appendix 3. Quality for analytical cross-sectional studies [16].**

|  | Were the criteria for inclusion in the sample clearly defined? | Were the study subjects and the setting described in detail? | Was the exposure measured in a valid and reliable way? | Were objective, standard criteria used for measurement of the condition? | Were confounding factors identified? | Were strategies to deal with confounding factors stated? | Were the outcomes measured in a valid and reliable way? | Was appropriate statistical analysis used? |
| --- | --- | --- | --- | --- | --- | --- | --- | --- |
| Campbell & McDowell, 2011 [38] | Y | Y | Y | NA | N | NA | Y | U |
| Do et al., 2020 [39] | Y | Y | Y | NA | Y | Y | Y | Y |
| Duffy et al., 2016 [40] | Y | Y | Y | NA | N | NA | Y | Y |
| Elhadi et al., 2021 [37] | Y | Y | Y | NA | N | NA | Y | Y |
| Gaumer et al., 2007 [41] | Y | Y | Y | NA | N | NA | U | Y |
| Gürdaş Topkaya & Kaya, 2015 [36] | Y | Y | Y | NA | N | NA | Y | Y |
| Hennemann et al., 2017 [35] | Y | Y | Y | NA | Y | Y | Y | Y |
| Kritsotakis et al., 2020 [42] | Y | Y | Y | NA | Y | Y | Y | Y |
| Olok et al., 2015 [43] | U | Y | Y | NA | N | NA | Y | U |
| Shiferaw & Mehari, 2019 [44] | U | Y | Y | NA | Y | Y | Y | U |
| Tesfa et al., 2021 [45] | Y | Y | U | NA | Y | Y | U | Y |
| Thapa et al., 2021 [46] | Y | Y | Y | NA | Y | Y | Y | Y |
| Vehko et al., 2019 [47] | Y | Y | Y | NA | Y | Y | Y | Y |

**Legend.** Y, Yes; U, Unclear when the information contained in the study was not sufficient; N, No; NA, Not Applicable given that the study had not the aim to assess cause-effect relationship, or an effect caused by an exposure. High quality: seven or eight “Yes”; Moderate quality: five or six “Yes”; Low quality: from one to four “Yes”.
