# Supplementary material for: Digital Health Competencies Among Health Care Professionals: Systematic Review
Source: J Med Internet Res. 2022 Aug 18;24(8):e36414. doi: 10.2196/36414 (PMC9437781; doi:10.2196/36414)
Supplement: Multimedia Appendix 4 [file jmir_v24i8e36414_app4.docx]

**Multimedia Appendix 4. Quality assessment for prevalence data studies [17].**

|  | Was the sample frame appropriate to address the target population? | Were study participants sampled in an appropriate way? | Was the sample size adequate? | Were the study subjects and the setting described in detail? | Was the data analysis conducted with sufficient coverage of the identified sample? | Were valid methods used for the identification of the condition? | Was the condition measured in a standard, reliable way for all participants? | Was there appropriate statistical analysis? | Was the response rate adequate, and if not, was the low response rate managed appropriately? |
| --- | --- | --- | --- | --- | --- | --- | --- | --- | --- |
| Brady & Knox, 2004 [24] | U | U | U | N | U | N | U | U | N |
| Hollander & Martin, 1999 [25] | U | U | U | N | U | U | U | U | Y |
| Kirchberg et al., 2020 [34] | U | N | U | Y | Y | Y | U | Y | N |
| Kleib & Nagle, 2018 [26] | Y | Y | Y | Y | Y | Y | Y | Y | Y |
| Kocher et al., 2021 [27] | Y | N | U | Y | N | Y | Y | Y | Y |
| Kujala et al., 2018 [28] | Y | Y | U | U | U | U | Y | U | U |
| MacLure & Stewart, 2015 [29] | Y | N | U | Y | Y | N | Y | U | U |
| Polhamus et al., 2000 [30] | U | U | U | N | U | U | U | U | U |
| Thomas & Ruttert, 2008 [31] | Y | Y | U | Y | Y | Y | Y | Y | N |
| van Houwelingen et al., 2019 [32] | Y | N | U | Y | Y | Y | Y | Y | Y |
| Zayapragassarazan & Kumar, 2016 [33] | U | N | U | U | Y | Y | Y | U | U |

**Legend.** Y, Yes; U, Unclear when the information contained in the study was not sufficient; N, No. High quality: eight or nine “Yes”; Moderate quality: six or seven “Yes”; Low quality: from one to five “Yes”.
