# Supplementary material for: Digital Health Competencies Among Health Care Professionals: Systematic Review
Source: J Med Internet Res. 2022 Aug 18;24(8):e36414. doi: 10.2196/36414 (PMC9437781; doi:10.2196/36414)
Supplement: Multimedia Appendix 5 [file jmir_v24i8e36414_app5.docx]

**Multimedia Appendix 5. Quality assessment for randomized control trial [18].**

|  | Was true randomization used for assignment of participants to treatment groups? | Was allocation to treatment groups concealed? | Were treatment groups similar at the baseline? | Were participants blind to treatment assignment? | Were those delivering treatment blind to treatment assignment? | Were outcomes assessors blind to treatment assignment? | Were treatment groups treated identically other than the intervention of interest? | Was follow up complete and if not, were differences between groups in terms of their follow up adequately described and analysed? | Were participants analysed in the groups to which they were randomized? | Were outcomes measured in the same way for treatment groups? | Were outcomes measured in a reliable way? | Was appropriate statistical analysis used? | Was the trial design appropriate, and any deviations from the standard RCT design (individual randomization, parallel groups) accounted for in the conduct and analysis of the trial? |
| --- | --- | --- | --- | --- | --- | --- | --- | --- | --- | --- | --- | --- | --- |
| Jouparinejad et al., 2020^49^ | Y | U | Y | NA | U | U | Y | Y | Y | Y | Y | Y | Y |
| Mastellos et al., 2018^48^ | Y | Y | Y | Y | N | Y | Y | Y | Y | Y | Y | Y | Y |

**Legend.** Y, Yes; U, Unclear when the information contained in the study was not sufficient; N, No; NA, Not Applicable. High quality: 12 or 13 “Yes”; Moderate quality: 10 o 11 “Yes”; Low quality: from one to nine “Yes”.
